# Social Perception and Interaction Database—A Novel Tool to Study Social Cognitive Processes With Point-Light Displays

**DOI:** 10.3389/fpsyt.2020.00123

**Published:** 2020-03-11

**Authors:** Łukasz Okruszek, Marta Chrustowicz

**Affiliations:** ^1^Social Neuroscience Lab, Institute of Psychology, Polish Academy of Sciences, Warsaw, Poland; ^2^Faculty of Psychology, University of Warsaw, Warsaw, Poland

**Keywords:** biological motion, communicative intentions, social perception, individual action, social interaction, point-light animations, emotion recognition

## Abstract

**Introduction:** The ability to detect and interpret social interactions (SI) is one of the crucial skills enabling people to operate in the social world. Multiple lines of evidence converge to indicate the preferential processing of SI when compared to the individual actions of multiple agents, even if the actions were visually degraded to minimalistic point-light displays (PLDs). Here, we present a novel PLD dataset (Social Perception and Interaction Database; SoPID) that may be used for studying multiple levels of social information processing.

**Methods:** During a motion-capture session, two pairs of actors were asked to perform a wide range of 3-second actions, including: (1) neutral, gesture-based communicative interactions (COM); (2) emotional exchanges (Happy/Angry); (3) synchronous interactive physical activity of actors (SYNC); and (4) independent actions of agents, either object-related (ORA) or non-object related (NORA). An interface that allows single/dyadic PLD stimuli to be presented from either the second person (action aimed toward the viewer) or third person (observation of actions presented toward other agents) perspective was implemented on the basis on the recorded actions. Two validation studies (each with 20 healthy individuals) were then performed to establish the recognizability of the SoPID vignettes.

**Results:** The first study showed a ceiling level accuracy for discrimination of communicative vs. individual actions (93% ± 5%) and high accuracy for interpreting specific types of actions (85 ± 4%) from the SoPID. In the second study, a robust effect of scrambling on the recognizability of SoPID stimuli was observed in an independent sample of healthy individuals.

**Discussion:** These results suggest that the SoPID may be effectively used to examine processes associated with communicative interactions and intentions processing. The database can be accessed via the Open Science Framework (https://osf.io/dcht8/).

## Introduction

Multiple lines of evidence indicate that encounters between other agents are preferentially processed by healthy individuals. Further, communicative interactions have been shown to be easily discriminated from other types of actions (1–3), gain preferential access to awareness (4), and are encoded as a single unit in working memory (5). Psychophysics experiments have also shown that healthy individuals are able to utilize top-down knowledge about the communicative gesture of one agent to predict both the type (6) and timing (7) of another agent's response. Furthermore, the processing of social interactions elicits widespread activation of the main “social brain” networks, compared to the individual actions of multiple agents (8–11). Importantly, these effects may be observed for both naturalistic full displays of agents (12–15) and minimalistic point-light displays of social interactions (8, 10, 11, 16). Developed by Johansson (17), point-light methodology limits the presentation of agents to a set of light-dots representing the head, limbs, and major joints of the agent's body. Despite the extremely limited amount of visual information presented via point-light displays (PLDs), this type of vignette has been shown to carry enough information to enable the recognition of an agent's action (18, 19), affective state (20), and a wide range of physical characteristics. Furthermore, point-light stimuli have also been used to investigate communicative intentions processing from both single (21) and dyadic displays (3).

Manera et al. (3) presented the Communicative Interaction Database (CID)—a set of 20 stimuli that presents dyadic interactions based on the stereotypical use of communicative gestures with point-light motion. CID stimuli have been used to examine both reflective (2) and reflexive (1) social cognitive processes in healthy individuals. Stimuli from the CID have also been used to create a multilingual task for studying communicative interaction recognition (2), which has been effectively applied to study social cognition across various clinical populations [patients with schizophrenia (22, 23), high functioning individuals with autism spectrum disorders (24), patients with temporal lobe epilepsy (25)]. Furthermore, CID stimuli have been applied to investigate the neural correlates of communicative interactions processing (10, 11). Additionally, as the CID database was created in adherence to the protocols used by Vanrie & Verfaillie (19), who presented a set of 22 non-communicative single-agent point-light actions, stimuli from both databases have previously been combined to obtain a broader spectrum of actions for studying the neural correlates of social interaction processing (11). However, the use of such a combination of stimuli from various datasets may be limited by several methodological factors (e.g., different actors presenting communicative vs. individual actions, varying length of the stimuli).

At the same time, given the widespread nature of social interactions (SI) processing across neural networks, a recent review of neural and behavioral findings in this area concluded that the development of SI localizers, which entail various types of social interaction vignettes, may facilitate research in this area (9). Studies based on static pictures of various types of social interactions have observed differential patterns of brain activity (14) and connectivity (26) in affective vs. cooperative interactions. Yet, due to the limited availability of point-light stimuli, previous studies on SI processing from PLDs either pooled various types of communicative interactions into one category [e.g. (8)] or presented only certain types of interactions (usually encounters based on the typical use of communicative gestures: (10, 11). Furthermore, it has been shown that communicative intentions may be differentially processed from the second person (receiver) and third person (observer) perspective (27). Thus, to address the second *person neuroscience* postulates (28), future studies should compare the processing of communicative intentions from the second person (single figure presenting gesture toward observer) and third person (displays of two agents acting toward each other) perspectives. The aim of the current project was to develop a database of point-light stimuli (Social Perception and Interaction Database; SoPID) that addresses the above listed issues by allowing for the creation of point-light animations with a wide range of communicative and individual actions, while flexibly manipulating the number of agents presented (one vs. two), the viewing perspective, and display options.

## Database Creation

### Pre-capturing Session

Two pairs of professional actors took part in the motion capture procedure. One dyad consisted of male actors and one of female actresses. During the pre-capturing session, actors were familiarized with the list of actions that were to be recorded. The list of situations to be recorded consisted of six categories, each with 5–10 situations (see Data Sheet 1 for a full list of the SoPID stimuli). For the communicative interactions (COM), each of the actors was asked to play both the person initiating the interaction via a communicative gesture (Agent A) and the person responding to the communicative gesture of the other agent (Agent B), thus producing two different takes on each COM situation. A short description of each action was provided to ensure that each dyad was enacting similar communicative intention and a similar behavioral response. To ensure the temporal synchronization of the animations, three sound signals were presented during each recording: first to signal the onset of the recording (played at T = 0 s.), second to signal the half-time of the recording (played at T = 1.5 s.), and third to signal the end of the recording (played at T = 3 s.). For animations that presented the sequential actions of both agents (e.g., Agent A asks Agent B to stand up, Agent B stands up), the actor playing Agent A was asked to start his action at T = 0, while the actor playing Agent B was asked to start responding at T = 1.5 s. The situations were rehearsed until both actors were able to perform them with the required timing. Moreover, to ensure the naturalistic yet expressive movement of the actors during the capturing sessions, a professional choreographer oversaw the actors' rehearsal during the pre-capturing session.

### Motion Capture

**Figure 1:**
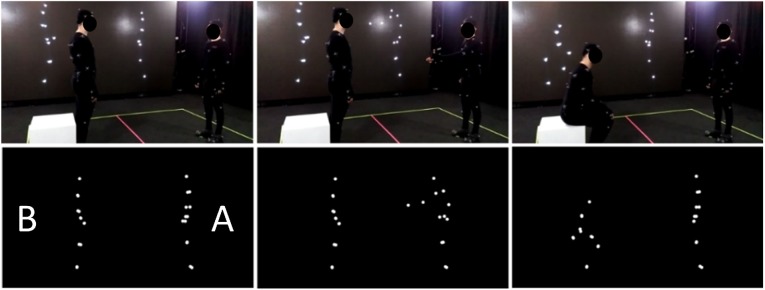
Original (**upper**) and PLD (**lower**) version of the item presenting communicative interaction from SoPID [A (on the right) asks B (on the left) to sit down; B sits down].

The motion capture session was performed via a motion-capture studio (*White Kanga* studio; Warsaw) using an OptiTrack (NaturalPoint, Corvallis, OR, USA) motion tracking system. Twelve OptiTrack Prime 13 cameras were utilized to record the movements of the actors at a 120 Hz rate. The actors wore 41 reflective spherical markers placed according to the OptiTrack Baseline+Hinged Toe system [full list and anatomical locations of the markers available at https://v20.wiki.optitrack.com/index.php?title=Baseline_%2B_Hinged_Toe,_with_Headband_(41)]. The motion capture room was a 7 x 7 meters square with a 3.8 meter high ceiling; a white line was painted on the floor of the motion capture room to mark each actor's subspace (7 x 3.5 meter). With the exception of enactments that included physical contact between the agents, the actors were asked to confine their actions within their subspaces. Similarly, most of the sequences were recorded with actors facing each other at a proximity of around 3 meters. At the beginning and end of each recording, the actors were asked to perform a T-pose (reference pose) at the central position of their subspace. Additional props were used for the sequences that included object-related actions (i.e., shovel, carton box, ax, saw, broom, glass, hammer, toothbrush, football, chair). No markers were used to tag the prop positions during the session, and thus the objects were not displayed in animations. For the stimuli that presented interaction between the agents (communicative interactions, happy/angry and synchronous interactive activity), the actions of both agents were recorded simultaneously to ensure that the response of one agent was congruent with the action of the other agent in terms of position, proxemics, and timing. Actions for object- and non-object related displays were recorded individually to minimize the potential effects of between-agents synchrony while performing the actions. Similarly, as during the pre-capturing session, sound cues were used to inform actors about beginning (T = 0 s.), middle (T = 1.5 s), and end (T = 3 s.) points of each three-second period. Furthermore, the point-light figures were previewed during the session to enable instantaneous re-takes for unsuccessful takes (Figure 1, upper).

### Data Processing

Data from the motion capture session were further processed using OptiTrack Motive 1.9 beta software. 2-D data from 12 cameras were used to obtain the 3-D coordinates of each marker. Skeleton models consisting of 13 bright dots corresponding to the head, arms, elbows, wrists, hips, knees, and ankles of each actor were animated. Data preprocessing included inspection of each of the recordings, data trimming to the period between the onset (T = 0 s.) and offset (T = 3 s.) of the action, and manual smoothing in case of any vibrating or fluttering movements. The preprocessed data were extracted to FBX files.

### Social Perception and Interaction Database

**Figure 2:**
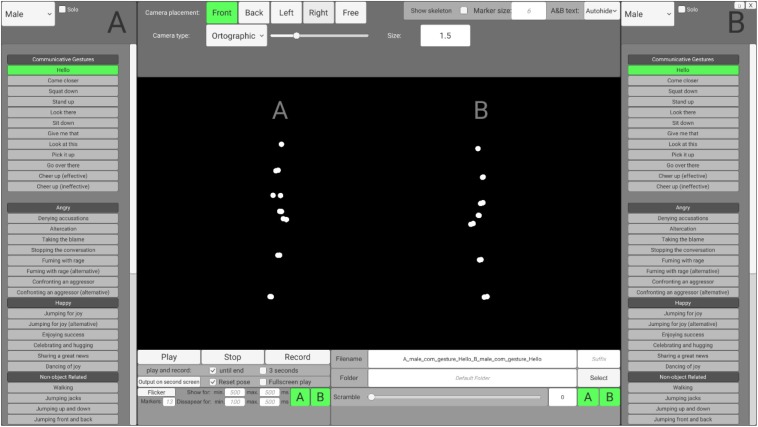
Social Perception and Interaction Database interface.

To enable users without programming skills to access and customize the stimuli according to their needs, preprocessed stimuli may be accessed via an interface that is based on the Unity engine (SoPID). The SoPID interface (which is visualized in the Figure 2) allows for modification of numerous stimuli characteristics and exports the customized stimuli to movie files (.mp4) using the FFmpeg codec. Overall, 64 different actions of each agent can be accessed via the SoPID and used to create experimental stimuli. Each of the recorded actions may be accessed either separately as a solo action or merged with a second action to produce a stimulus presenting a pair of agents. This way, the SoPID allows for a wide range of animations presenting a single agent's communicative or individual actions to be produced. It also allows for the actions of two agents to be combined into either congruent (by selecting one out of four Agent A's “Communicative Gestures” and any of the corresponding responses of Agent B or by using a combination of either “Happy,” “Angry,” or “Synchronous Interactive Activity” actions) or incongruent (e.g., by mixing Agent A's communicative action with a non-corresponding action of Agent B) social interactions or parallel individual actions of agents. The whole list of actions available in the SoPID is presented in Supplementary Table 1.

The SoPID interface also allows for flexible adjustment of camera position. Four standard camera positions may be selected, with the “Front” position corresponding to a 270 degree display from the CID (Agent A on the left and Agent B on the right) with the camera being placed on the middle line between the agents, at a height of one meter and 15 meters from the agents. Furthermore, by using the “Free” option, both the camera placement (x—left/right; y—up/down; z—closer/further; values in meters) and rotation (x—up/down; y—left/right; z—horizontal/vertical; values in degrees) can be fully customized. Both ortographic (with no depth cues—all points are same size) and perspective (containing depth cues—parts of the actor that are further from the observer are depicted by smaller points) projections may be used to manipulate the availability of depth cues in the animations. Additionally, marker size may be changed (“Marker size,” values in centimeters) to modify the agents' appearances and stick figures can be created instead of point-light displays (“Show skeleton”). Finally, two standard modifications that are commonly used in point-light studies can be applied directly via the SoPID. First, by using the “Flicker” option, the visual availability of the stimuli may be limited by selecting the maximal number of simultaneously displayed markers (0–13) and the time range for marker display/disappearance. Markers are flickered by randomly assigning the onset and offset time values separately for each marker with regard to the time range provided by the user. In addition, by using the “Scramble” option, the initial spatial position of each marker can be spatially scrambled. Scrambling is applied by randomly drawing one out of three dimensions for each marker and relocating its initial position by X centimeters from its initial position in the selected direction (e.g., 100% scrambling moves each marker by one meter in either the x, y or z dimension). “Flicker” and “Scramble” can be applied to both agents or selectively to each agent. The database, as well as raw motion capture files, can be accessed via the Open Science Framework (https://osf.io/dcht8/).

## Methods

To examine the recognizability of the presented actions and the effectiveness of the scrambling mechanism, two SoPID validation studies were performed. The aim of Study 1 was to investigate the detection of communicative intentions and the recognition of specific actions of agents across a wide range of social interactions and parallel non-communicative actions included in SoPID. The goal of Study 2 was to examine the effectiveness of the display manipulation procedures (in particular biological motion scrambling procedure) implemented in SoPID, by comparing the recognizability of human motion under various levels of scrambling.

### Stimuli

#### Study 1

Fifty-seven animations presenting the actions of two agents were created using the SoPID (perspective camera with FoV = 10°, camera position = front, and marker size = 6). Six types of stimuli were presented throughout the study: Communicative gestures [COM, 10 animations: “Hello” (Female 2 as Agent A); “Come closer” (Male 1 as Agent A), “Squat down” (F2), “Stand up” (M1), “Look there” (F1), “Sit down” (M2), “Give me that” (F2), “Look at this” (M1), “Pick it up” (F2), “Go over there” (M2)]; Angry exchanges (Angry, 5 animations: “Denying accusations,” “Taking the blame,” “Stopping the conversation,” “Fuming with rage,” “Confronting an aggressor (alternative)”); Happy exchanges (Happy: 5 animations: “Jumping for joy,” “Enjoying success,” “Celebrating and hugging,” “Sharing a great news,” “Dancing of joy”); Non-object related parallel individual actions (NORA, 10 animations: “Walking,” “Jumping jacks,” “Jumping up and down,” “Jumping front and back,” “Arm waving,” “Hip swinging,” “Torso twist,” “A-Skip,” “Squat down,” “Lateral step,” “Lateral kick”); Object related parallel individual actions (ORA, 9 animations: “Shoveling,” “Lifting the box,” “Chopping wood,” “Sawing,” “Digging,” “Sweeping the floor,” “Drinking,” “Hammering a nail,” “Brushing teeth”); and Synchronous interactive activity of two agents [SYNC, 8 animations: “Dancing” (M/F), “Fencing” (M), “Football” (F), “Throwing the ball” (M), “Boxing” (F), “Kickboxing” (M/F)]. To ensure that a similar number of stimuli were presented for each category and to increase the comparability of recognition accuracy levels across the categories, two stimuli (one with male and one with female actors) were created for each situation from the Angry and Happy categories. ORA and NORA movies were created by merging the displays of two different actions performed by two same-sex actors. Displays of each set of actions with either male or female actors were included, thus producing 11 NORA and 9 ORA movies in total.

#### Study 2

Twenty movies [“come closer” (F), “squat down” (M), “stand up” (M, F), “go over there” (F), “altercation” (M, F), “jumping for joy” (F), “denying accusations” (M), “jumping for joy (alternative)” (M), “walking” (F), “lateral kick” (F), “hip swinging” (M), “A-skip” (M), “squat down” (M), “lifting the box” (F), “sweeping the floor” (F), “brushing teeth” (F), “chopping wood” (M), “digging” (M)] presenting the action of a single agent (Agent A in case of COM, Angry and Happy) were created from the SoPID (ortographic camera (size = 1.5), camera position = right, and marker size = 6). Each animation was rendered at four scrambling levels: 0, 15, 30, and 100%. Thus, 80 animations were presented during the experimental procedure.

### Participants

Participants for each of the studies were recruited from the students of Warsaw-based universities. All of the participants were right-handed. Participants were tested individually, and had not participated in point-light experiments prior to the examination. Twenty participants (9M/11F; 25.9 ± 9.1 yrs. old) completed Study 1, while 20 participants (10M/10F; 24.2 ± 7.7 years old), who did not participate in Study 1, completed Study 2.

### Apparatus and Procedures

#### Study 1

Each stimulus was presented twice, after which participants were asked to: (1) classify whether the presented action was an interaction (behavior of one agent affects the behavior of the other) or not by responding to the response screen with two options (Interaction vs. No interaction), and (2) to provide a verbal description of the actions of the agents (which was written down by the experimenter). The order of stimuli presentation was pseudorandomized to avoid subsequent presentation of more than two stimuli from the same category. The paradigm was programmed using NBS Presentation 20, and the whole procedure took ~1 h. Verbal descriptions provided by the participants were scored by a rater who did not participate in data collection. Spontaneous descriptions for COM, SYNC, Happy, and Angry were scored in a dichotomic manner (2 points for a correct verbal description vs. 0 points for an incorrect description). Accuracy for ORA and NORA stimuli was calculated by scoring one point for each correctly recognized action from male and female presentations (0–2 points). For interaction vs. individual actions classification, COM, Angry, Happy and SYNC were treated as falling into the category “interaction”, while ORA and NORA were treated as “individual actions.” Two items (“Dancing for joy” and “Fuming with rage”) without any explicit communicative cues were discarded from this part of the analysis.

#### Study 2

Upon presentation of each animation, participants were asked to indicate whether the presented animation resembled human motion. Completion of the whole experimental procedure took approximately 20 min.

### Statistical Analysis

#### Study 1

To examine between-category differences in accuracy levels, one way ANOVAs with Type of animation (six levels) were performed separately for interaction recognition and spontaneous identification of actions.

#### Study 2

The number of stimuli classified as “human” at each scrambling level was compared to examine the effectiveness of the scrambling procedure. The results were analyzed using rmANOVA with the within-subject factor Scrambling (4 levels: 0, 15, 30, 100%).

## Results

### Study 1

Behavioral accuracies for each type of the task are presented below in Table 1.

**Table 1:** Behavioral accuracy for recognition of communicative intentions and identification of specific actions in Study 1 (mean ± standard deviation is given for each category).

	**Angry**	**Happy**	**COM**	**SYNC**	**NORA**	**ORA**
**Study 1 results**						
Recognition of communicative intentions (%)	95 ± 9	89 ± 13	95 ± 10	91 ± 11	94 ± 10	93 ± 8
Identification of specific action (%)	81 ± 12	92 ± 11	78 ± 12	96 ± 6	95 ± 5	67 ± 13

#### Recognition of Communicative Intentions

No between category differences were observed for classifying actions as either communicative or individual [*F*_(5, 15)_ = 1.3; n.s., $\eta_{\text{p}}^{2}$ = 0.07], with ceiling level recognition for all types of items. As ceiling effects were observed for most of the categories in Study 1, we re-examined the results with a non-parametric Friedman test of differences among repeated measures, which provided a Chi-square value of 8.94 (*p* > 0.05).

#### Identification of Specific Action

A main effect of category was observed for the accuracy of identification of specific actions [*F*_(5, 15)_ = 23.9, p < 0.001, $\eta_{\text{p}}^{2}$ = 0.56]. Further investigation of this effect revealed the highest recognition for SYNC, NORA and Happy, each of which were identified at higher level than Angry and COM. Furthermore, actions from all of the categories were identified more accurately than ORA. As in the case of Study 1, we re-examined these non-normally distributed variables with the Friedman test, which provided a Chi-square value of 49.24 (*p* < 0.001).

### Study 2

One participant with results over three standard deviations from the mean value in two conditions (0 and 100%) was excluded from the analysis. A robust effect of scrambling was observed [*F*_(3, 16)_ = 400.9; *p* < 0.001, $\eta_{\text{p}}^{2}$ = 0.96]. Unscrambled stimuli were classified as human motion significantly more often than 15, 30, and 100% scrambled motion. Similarly, 15% stimuli were classified as human motion more often than 30 and 100% scrambled displays, and 30% scrambled stimuli were classified as human more often than 100% scrambled displays. All of the contrasts were significant at *p* < 0.001. Similarly to Study 1, as a non-normal distribution of results was observed for 0 and 100% scrambled motion classification, we re-examined the results with the Friedman test and found a significant (*p* < 0.001) effect with a Chi-square of 55.68. The percentage of the stimuli classified as a human motion at each scrambling level is presented in Table 2.

**Table 2:** Percentage of stimuli classified as a human motion for various levels of scrambling in Study 2 (mean ± standard deviation is given for each category).

**Scrambling level**	**0%**	**15%**	**30%**	**100%**
**Study 2 results**				
Percentage of stimuli classified as a human motion	98 ± 3	68 ± 18	20 ± 13	3 ± 5

## Discussion

The present paper describes the Social Perception and Interaction Database, a novel set of point-light displays that enables study of the processing of a wide range of communicative and individual actions from single-agent and two-agent vignettes. The SoPID includes 32 animations presenting various types of social interactions between two agents, including standard use of communicative gestures (COM), synchronous interactive physical activity (SYNC) and affective exchanges (either Happy or Angry), as well as 20 animations of each actor performing either object- (ORA) or non-object-related (NORA) individual actions. Furthermore, by performing two validation studies, we established that SoPID vignettes elicit similar effects to those previously described in studies on intention and emotion processing from PLDs.

Previous studies that used the CID database showed high accuracy in recognition of communicative vs. individual actions in healthy individuals (2, 29). Similarly, we observed a ceiling level accuracy for classifying stimuli as either communicative or individual across the six categories of stimuli included in the first of the validation studies (ranging from 89% for Happy to 95% for COM). Furthermore, the accuracy of identification of specific communicative actions from the COM category (78% ± 12%) was at a similar level as previously reported for the multilingual CID task [74% ± 18%; (29)]. Interestingly, more accurate identification of specific actions was observed for three other categories of stimuli included in the study (Happy, NORA, SYNC). This result may be linked to the fact that both NORA and SYNC stimuli presented physical activity that is usually associated with whole-body motion (e.g., jumping or kick-boxing), which may have higher salience compared to the more restricted actions (e.g., hand gestures) presented across other categories. Thus, (1) as a salient movement may have been easier to classify and (2) communicative gestures need higher-order processing, and attribution of intent, both of these aspects may have contributed to the better accuracy for NORA and SYNC. Increased recognition of positively-valenced social exchanges (Happy) compared to neutral communicative interactions (COM) and negatively-valenced social exchanges (Angry) is congruent with previous findings showing that the positive emotional valence of stimuli facilitates biological motion processing from single (30) and dyadic (31) point-light displays. Finally, we observed that while object-related individual actions were identified less accurately than any other type of SoPID animations (67% ± 13%), their recognition rate was at a similar level as the recognition rates of individual actions from the well-established stimuli set by Vanrie et al. (19), which reported a mean accuracy rate of 63% for a set of individual point-light actions in 11 observers producing spontaneous descriptions of the animations. In a second validation study, a robust effect of the scrambling mechanism implemented in SoPID was found: unscrambled and 100% scrambled stimuli were almost unanimously categorized as, respectively, human and non-human motion. Furthermore, the more subtle effects of scrambling were also observed: a significant portion of the 15% scrambled stimuli were classified by participants as resembling human motion, while a large majority (80%) of the 30% scrambled stimuli were classified as non-human motion.

These results suggest that the SoPID stimuli may be effectively used in a wide range of experiments examining both basic (e.g., recognition of biological vs. scrambled motion) and higher-order (e.g., recognition of communicative intentions of affective states from PLDs) processing of biological motion. Moreover, a recent review of the findings on emotion and intention processing from biological motion in psychiatric disorders (32), has concluded that disorder-specific social cognitive biases (e.g., negativity bias in depression, abnormal threat perception in anxiety) may be effectively elicited by biological motion vignettes. The use of recorded situations during which two or more actors interact with each other is currently the primary method for social cognitive assessment of communicative interactions processing (33, 34). However, patients with neuropsychiatric disorders have been shown to present decreased ability to process a wide range of social signals (e.g., facial expressions, non-verbal prosody, bodily movements), which need to be successfully integrated to correctly process such complex situations. Thus, between-group differences observed in studies based on paradigms that utilize full-displays of actors to examine communicative interactions processing in neuropsychiatric populations may be affected by other perceptual issues or potentially distracting elements of visual displays. Decreased recognition of affective states and/or communicative intentions from point-light displays have previously been documented in individuals with ASD (35), patients with schizophrenia (36), affective disorders (37), neurodegenerative diseases (38) and temporal lobe epilepsy (25). Furthermore, previous studies that used CID stimuli have provided evidence that a double dissociation between explicit and implicit processes associated with communicative interactions detection may be observed in two neuropsychiatric populations (23, 24). Okruszek et al. (23)observed that patients with schizophrenia, while being less accurate in explicitly interpreting communicative interactions presented with point-light displays, are able to use the communicative action of one agent to predict the response of another agent during an implicit task (“interpersonal predictive coding”). However, the reverse pattern (intact explicit recognition of actions, but no interpersonal predictive coding during an implicit task) was observed in high-functioning individuals with ASD (24). At the same time, the scope of the previous research in this area, due to the limited availability of the stimuli, has been limited to recognition of intentions from standard communicative gestures from either single (38) or dyadic (22, 24, 25) displays. Therefore, use of the SoPID may extend the area of investigation of future studies to neuropsychiatric populations, by enabling the examination of behavioral and neural responses to a wide range of individual actions and communicative actions with or without emotional content.

Investigation of the behavioral and neural correlates of social interactions processing has been the focus of increasing interest in recent years (9). Additionally, a framework integrating current knowledge about the factors shaping the perception of social interactions has recently been proposed [Integrative Model of Relational Impression Formation, IMRIF; (39)]. The IMRIF emphasizes that accuracy of the perception of social interactions is determined by four main types of attributes: (1) content attributes (factors related to the specifics of the interaction which is being perceived), (2) target attributes (characteristics of the interacting agents), (3) perceiver attributes (characteristics of the person perceiving the interaction), and (4) context attributes (specific circumstances under which an interaction is being perceived) (39). By providing a rich source material that can be further customized in multiple ways, the SoPID may be effectively used to examine a wide range of research questions regarding the factors impacting SI processing that have been suggested by IMRIF.

Firstly, by including a wide range of actions from various semantic categories and allowing users to create stimuli by combining the actions of both agents, both within each category and between the categories, a wide range of novel stimuli can be created to study the impact of the content attributes on social interaction processing. In addition, by enabling the congruency of the actions in dyadic displays to be manipulated to create both typical and novel ambiguous or paradoxical situations (e.g., agent B performs an action that is opposite to the request of agent A). Secondly, target attributes can also be changed by either modifying the presentation of the agents (e.g., point-light agents vs. stick figures) or, as the SoPID includes actions produced by four different actors (two male and two female), by presenting the same situations involving different agents. Finally, by enabling one to manipulate the observer's visual perspective and the presence of the second agent's response, contextual factors impacting the SI processing can also be studied. For example, by presenting the same stimuli from a second- and third-person perspective, the impact of the participant vs. observer role for communicative intentions processing can be examined. It has been shown that communicative intentions directed toward the participant (second person perspective) elicit larger activity within the crucial nodes of the mentalizing network (medial prefrontal cortex, mPFC) and mirroring (bilateral premotor cortex) compared to the observation of the same communicative intentions observed from the third person perspective (27). Similarly, the specific impact of the egocentric (second-person) vs. allocentric (third-person) perspective on neural activity elicited by coverbal gestures was observed in the anterior cingulate region (40). These findings, which suggest that neural computations supporting communicative intention processing may be affected by the observer vs. participant point of view, emphasize the importance of further investigating the role of contextual factors in communicative interactions processing.

The necessity of developing new tasks to study the factors impacting third party encounter processing has recently been stressed (9). By introducing a tool that enables manipulation of SI content, target characteristics and contextual factors, the SoPID allows for flexible creation of stimuli to develop novel tasks for behavioral and neuroimaging research and to address novel research hypotheses.

## Data Availability Statement

The datasets analyzed for this study can be found in the OSF-HOME repository - https://osf.io/dcht8/.

## Ethics Statement

The studies involving human participants were reviewed and approved by Institute of Psychology, PAS. Written informed consent for participation was not required for this study in accordance with the national legislation and the institutional requirements.

## Author Contributions

ŁO contributed conception and design of the study, performed the statistical analysis, and wrote the first draft of the manuscript. MC collected the data and wrote a section of the manuscript. All authors contributed to manuscript revision, read, and approved the submitted version.

### Conflict of Interest

The authors declare that the research was conducted in the absence of any commercial or financial relationships that could be construed as a potential conflict of interest.
