# Supplementary material for: Social Perception and Interaction Database—A Novel Tool to Study Social Cognitive Processes With Point-Light Displays
Source: Front Psychiatry. 2020 Mar 11;11:123. doi: 10.3389/fpsyt.2020.00123 (PMC7078367; doi:10.3389/fpsyt.2020.00123)
Supplement: Supplementary file 1 [file Table_1.pdf]

Supplementary Table 1. Percentage of correct responses for task 1 (classification of actions) and task 2 (identification of specific action) for each of the Social Perception and Interaction Database animations.

**Communicative interactions**			
NAME	DESCRIPTION	IDENTIFICATION OF SPECIFIC ACTION (% correct)	CLASSIFICATION OF ACTIONS (COMMUNICATIVE vs INDIVIDUAL) (% correct)
“Hello”	A waves to B, B waves back to A.	70	90
“Come closer”	A asks B to come closer, B comes closer.	100	100
“Squat down”	A asks B to squat down, B squats down.	70	90
“Stand up”	A asks B to stand up, B stands up.	100	100
“Look there”	A points something in the sky, B looks at it.	42.5	95
“Sit down”	A asks B to sit down, B sits down.	100	100
“Give me that”	A points something to B, B lifts it and gives it to A.	40	95
“Look at this”	A points something on the floor to B and squats down to look at it, B squats down next to A to look at it.	100	100
“Pick it up”	A points something on the floor to B, B picks it up and hides in a pocket.	85	95
“Go over there”	A asks B to go away, B turns around and leaves.	72.5	85
“Cheer up (effective)”	A squats on the floor and is crying, B comes closer to A and puts hand on A’s shoulder, A stands up.	NA	NA
“Cheer up (ineffective)”	A squats on the floor and is crying, B comes closer to A and puts hand on A’s shoulder, A shakes B’s hand off.	NA	NA
**Emotional interactions: Angry exchanges**			
“Denying accusations”	A accuses B of something, B denies it.	87.5	97.5

“Altercation”	A and B quarrel with each other.	NA	NA
“Taking the blame”	A accuses B of something, B takes blame.	97.5	95
“Stopping the conversation”	A accuses B of something, B says ‘Stop’.	55	90
“Fuming with rage”	A and B jump furiously.	50	NA
“Confronting an aggressor”	A comes at B, B stops A with a hand.	97.5	97.5
**Emotional interactions: Happy exchanges**			
“Jumping for joy”	A and B start jumping for joy.	93.75	85
“Enjoying success”	A and B are excited and they give each other high five.	87.5	92.5
“Celebrating and hugging”	A and B jump for joy and hug each other.	100	97.5
“Sharing a great news”	A tells some good news to B, B starts jumping.	85	80
“Dancing of joy”	A and B dance to celebrate some success.	92.5	NA
**Non-object related individual actions**			
“Walking”	Agent walks towards the camera.	92.5	-
“Jumping jacks”	Agent does three jumping jacks.	97.5	-
“Jumping up and down”	Agent jumps up and down.	97.5	-
“Jumping front and back”	Agent does forward jump toward the camera and then immediately jumps backward.	92.5	-
“Arm waving”	Agent swings arms back and forth.	100	-
“Hip swinging”	Agent does hip swinging exercise.	100	-
“Torso twist”	Agent does torso twist exercise.	100	-
“A-Skip”	Agent does A-skip exercise.	95	-

“Squat down”	Agent does a squat.	95	-
“Lateral step”	Agent does a step to the right and then a step to the left while facing the camera.	90	-
“Lateral kick”	Agent does a side-kicks while facing the camera.	90	-
**Object related individual actions**			
“Shoveling”	Agent moves something with a shovel.	72.5	-
“Lifting the box”	Agent squats down and lifts up a box.	45	-
“Chopping wood”	Agent chops the wood with an axe.	65	-
“Sawing”	Agent uses hand saw to cut wood.	12.5	-
“Digging”	Agent is digging a hole with a shovel.	97.5	-
“Sweeping the floor”	Agent is sweeping a floor with a broom.	97.5	-
“Drinking”	Agent raises a glass and drinks something from it.	70	-
“Hammering a nail”	Agent is hammering a nail.	82.5	-
“Brushing teeth”	Agent is brushing teeth with right hand.	50	-
**Synchronous activity of two agents**			
“Dancing”	A and B perform a dance routine.	100	67.5
“Fencing”	A and B practice fencing.	95	95
“Football”	A and B play football.	100	100
“Throwing the ball”	A throws the ball to B, B catches and throws it back to A.	90	100
“Boxing”	A and B throw punches at each other while boxing.	100	100
“Kickboxing”	A and B perform a kickboxing routine.	92.5	97.5
