# Supplementary material for: Social Perception and Interaction Database—A Novel Tool to Study Social Cognitive Processes With Point-Light Displays
Source: Front Psychiatry. 2020 Mar 11;11:123. doi: 10.3389/fpsyt.2020.00123 (PMC7078367; doi:10.3389/fpsyt.2020.00123)
Supplement: Supplementary file 2 [file Data_Sheet_1.ZIP › Stimuli examples/S2. List of stimuli.docx]

| **Name** | **Category** | **Situation** |
| --- | --- | --- |
| S1.1. | Communicative – dyadic | “Come closer” (Male 1 as Agent A) |
| S1.2. | Angry – dyadic | “Stopping the conversation” |
| S1.3. | Happy – dyadic | “Celebrating and hugging” |
| S1.4. | Non-object related actions - dyadic | Agent A - “Walking”  Agent B – “Jumping jacks” |
| S1.5. | Object related actions – dyadic | Agent A - “Brushing teeth”  Agent B – “Lifting the box” |
| Synchronous activity | Synchronous activity – dyadic | “Fencing” (M) |
| S2.1. | Single – non-scrambled | “Hello” |
| S2.2. | Single – 15% scrambled | “Hello” |
| S2.3. | Single – 30% scrambled | “Hello” |
| S2.4. | Single – 100% scrambled | “Hello” |
